# Dead Legs: A Case of Bilateral Leg Paralysis

**DOI:** 10.5811/cpcem.2017.5.34091

**Published:** 2017-10-03

**Authors:** Faith Quenzer, Joel Stillings, Jacqueline Le

**Affiliations:** Desert Regional Medical Center, Department of Emergency Medicine, Palm Springs, California

## Abstract

Aortoiliac occlusive disease (AOD) is a rare presentation of thrombosis of the abdominal aorta. Also known as Leriche syndrome, its classic description entails claudication of the buttocks, thighs, and calves, absent femoral pulses, and impotence. AOD risk factors include smoking, hypertension, hyperlipidemia, diabetes, chronic renal insufficiency, and hypercoagulopathy. Ischemic complications of gastrointestinal malperfusion, renal infarction, and paralysis secondary to spinal cord ischemia are also noted. This case describes AOD complicated by a Stanford Type B aortic dissection leading to multi-system organ failure. A brief review of the literature further elucidates the key risk factors in identifying and treating Leriche syndrome.

## INTRODUCTION

Aortoiliac occlusive disease (AOD) was first described by Dr. Rene Leriche in 1940. Also known as Leriche syndrome, AOD is a rare presentation of thrombosis of the abdominal aorta. Leriche’s classic triad of claudication of the buttocks, thighs, and calves, absent femoral pulses, and impotence are all consistent with thrombotic obliteration at the end of the aorta.^1^,^2^ Found mostly in males in their third to sixth decades of life, the risk factors for AOD include smoking, hypertension, hyperlipidemia, and diabetes.^3^ Ischemic complications of AOD include gastrointestinal malperfusion, renal infarction, and paralysis secondary to spinal cord ischemia.^3^ In conjunction with the physical examination, computed tomography angiography (CTA) and point-of-care ultrasound help confirm the diagnosis. Operative management consists of aortobifemoral bypass grafting, endovascular intervention, or angioplasty and stenting.

## CASE REPORT

A 69-year-old man with a past medical history of untreated hypertension, chronic back pain, and heavy smoking was brought in by ambulance to a community emergency department (ED) for severe bilateral leg pain and paraplegia. He had been unable to ambulate and remained lying on the floor for the prior three days. The patient also admitted to chronic low back pain that had worsened over the preceding week and not improved with his usual pain medications of naproxen and aspirin. He had no other complaints and denied any fevers or chills.

The patient’s initial vital signs were as follows: temperature 36.6 degrees Celsius, blood pressure 157/84 mmHg, heart rate 95 beats/min, respiratory rate 38 breaths/min, oxygen saturation 94% on room air. On physical examination, the patient appeared cachectic and chronically ill. His cardiac exam revealed a regular heart rate and rhythm without murmur. He had no palpable femoral, popliteal, or dorsalis pedis pulses bilaterally. A Doppler ultrasound performed at bedside further demonstrated a lack of pulses bilaterally from the femoral to the dorsalis pedis arteries. Abdominal examination showed a scaphoid, soft, non-tender abdomen without a pulsatile mass. There was no midline spinal tenderness or step-offs on musculoskeletal examination. Neurological evaluation demonstrated complete sensory loss from T10 and ending on the S1dermatome bilaterally. He had 5/5 motor strength in both upper extremities and 0/5 strength in hip flexion and extension as well as ankle dorsiflexion and plantar flexion bilaterally. The dermatological examination of the lower extremities revealed pale, cold, and mottled-appearing skin from the hips to the ankles bilaterally.

Initial laboratory data for the patient revealed white blood cells (WBC) 26.9 x10^9^/L with neutrophil count 88.8%, hemoglobin 14.3 g/L, hematocrit 44.4%, and platelets 288 x 10^9^/L complete metabolic panel showed the following: sodium (Na) 127 mmol/L, potassium (K) 7.3 mmol/L, chloride (Cl) 99 mmol/L, carbon dioxide (CO_2_) 18 mmol/L, blood urea nitrogen (BUN) 82 mg/dL, creatinine (Cr) 4.4 mg/dL, glucose 123 mg/dL, alanine transaminase 288 units/L, aspartate transaminase 1,372 units/L, albumin 3.4g/dL, alkaline phosphatase 86 units/L, direct bilirubin 1.0 mg/dL, indirect bilirubin 0.2 mg/dL, and total bilirubin 1.2 mg/dL. Prothrombin time, international normalized ratio, and partial thromboplastin time were 11.0, 1.0, and 26.9 seconds, respectively. Lactic acid was 3.36 mmol/L and creatinine kinase was 111,693 units/L. Troponin was also elevated at 3.850 ng/mL, and the electrocardiogram showed diffuse and deep T-wave inversions in the inferior, anterior, and lateral leads.

**Image 1:**
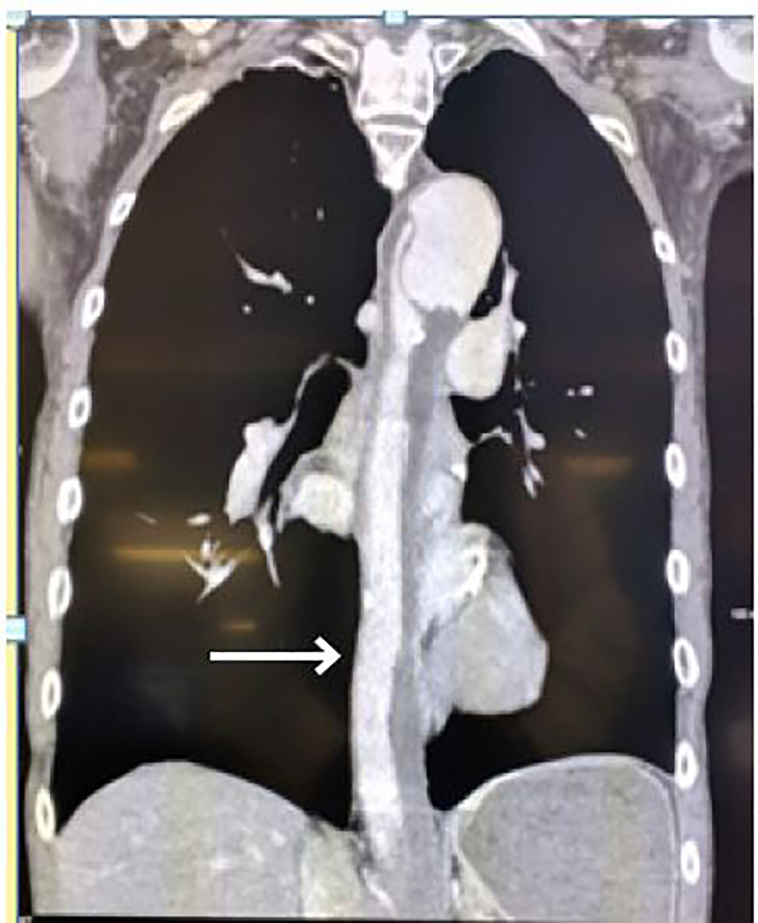
Computed tomography of the chest in coronal view demonstrating a Stanford Type B aortic dissection (arrow).

**Image 2:**
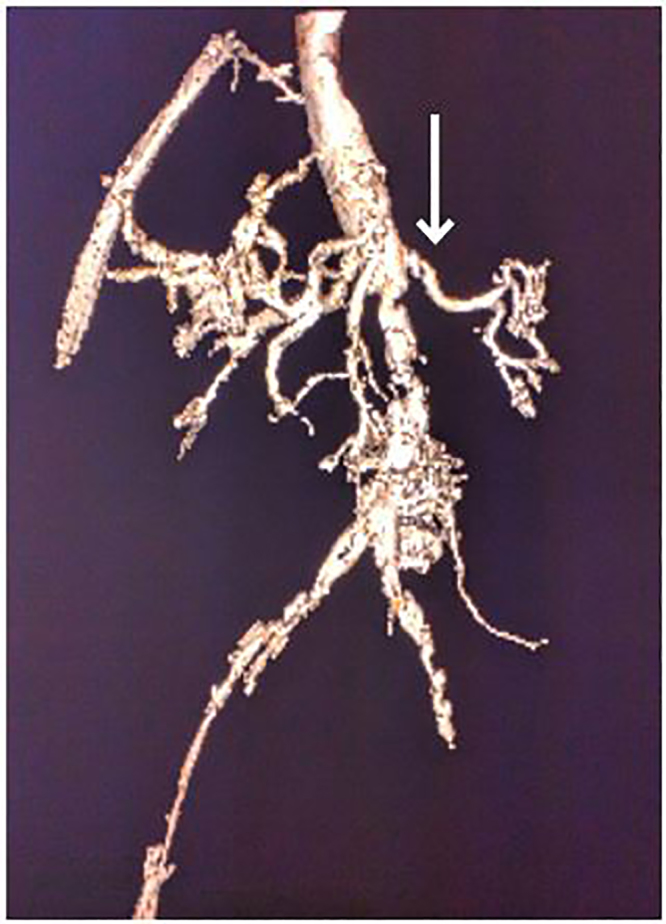
Computed tomography angiography of the abdominal aorta and bilateral lower extremities with runoff in three-dimensional reconstruction. Severe narrowing at the origin of the left renal artery is visible (arrow).

**Image 3:**
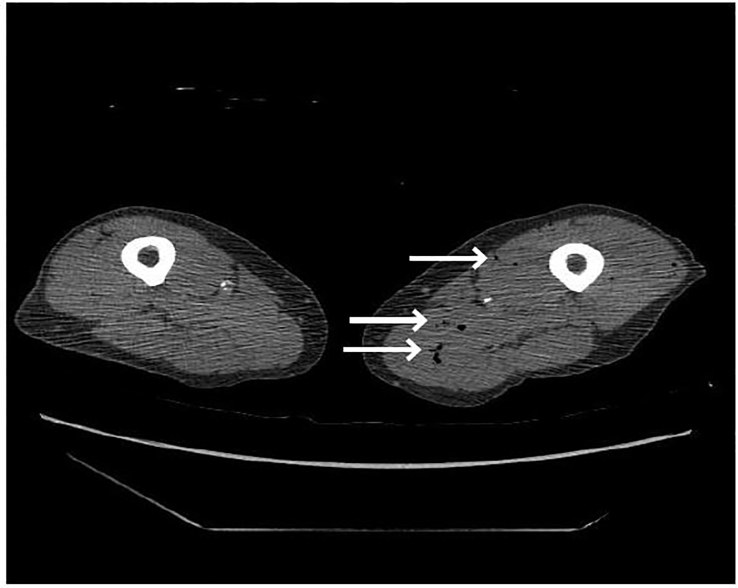
Computed tomography angiography of bilateral lower extremities displaying intramuscular gas, signifying myonecrosis in both thighs (arrows).

Point-of-care ultrasound of the abdominal aorta revealed a linear hyperechoic shadow in the aorta. Chest radiograph displayed a mildly tortuous ectatic aorta and evidence of chronic obstructive pulmonary disease (COPD). CT of the chest demonstrates a Stanford Type B aortic dissection (Image 1). The dissection starts from the subclavian and extends well into the infrarenal region of the abdominal aorta with significant mural thrombosis. On three-dimensional reconstructed images of the CTA abdominal aorta and bilateral lower extremities with runoff, significant stenosis is visible in both renal arteries, especially in the left (Image 2). The right lower extremity demonstrates near-complete occlusion of the right common iliac artery and both internal and external iliac arteries. The popliteal artery and arteries below the right knee are occluded. No blood flow to the right lower extremity is noted. The left lower extremity also demonstrates severe narrowing with moderate thrombosis in the iliac arteries with complete occlusion of the left external iliac artery. The left popliteal artery is likewise occluded, and no blood flow to the left lower extremity is visible. In addition to the above findings, intramuscular gas is seen in the thighs bilaterally, raising suspicion for myonecrosis (Image 3).

**CPC-EM Caspule**

What do we already know about this clinical entity?

Leriche syndrome is an aortoiliac occlusion that classically presents as claudication of the thighs, calves, and buttocks, impotence, and pulselessness of the legs.

What makes this presentation of disease reportable?

This is the first reported presentation of Leriche syndrome that involved a Stanford Type B aortic dissection.

What is the major learning point?

Aortoiliac occlusion (Leriche syndrome) should still be in the differential diagnosis in a patient with bilateral leg paralysis and decreased pulses bilaterally.

How might this improve emergency medicine practice?

An awareness and understanding of Leriche syndrome may potentially improve morbidity and mortality of this rare yet devastating disease.

In the ED, the patient was aggressively resuscitated with an initial two-liter bolus of normal saline. A central venous catheter was inserted in the left internal jugular vein and the patient was started on an intravenous (IV) esmolol drip. He also received IV heparin, calcium chloride, sodium bicarbonate, insulin, glucose, and morphine during his ED stay. Shortly thereafter, the patient was taken for emergent dialysis in the intensive care unit (ICU) to further correct his electrolyte abnormalities, renal failure, and rhabdomyolysis prior to operative treatment. The laboratory abnormalities were partially corrected to a WBC of 17.5 with neutrophil count of 79.7%, K of 6.6, BUN of 87 and Cr at 3.6. The patient subsequently underwent open bilateral iliofemoral aortic popliteal thrombectomy, stent placement in the abdominal aorta and bilateral iliac arteries and bilateral femoral endarterectomy and patch angioplasty. While in the operating room, the patient became hypotensive and bradycardic and eventually suffered cardiopulmonary arrest. Despite a brief return of spontaneous circulation after resuscitative efforts, the patient died shortly thereafter in the ICU.

## DISCUSSION

Leriche syndrome is a rare and potentially devastating disease. It is often an advanced and indolent manifestation of thrombotic accumulation. Reported mortality rates of AOD range from 25% to 75%.^1^,^2^ The classic triad Leriche described includes claudication of the buttocks, thighs, and calves, absent femoral pulses, and impotence. These traits were found in 73% of men with aortoiliac occlusion. From 45% to 64% of patients will complain of claudication in the buttocks, thighs, and/or calves. Other clinical features include paralysis, pallor, or mottled appearance of the lower extremities. Massive ischemic complications have been observed in AOD, such as gastrointestinal hypoperfusion, renal infarction, and paralysis secondary to spinal cord ischemia. All of these symptoms correlate well to the anatomical region that has been occluded.^2^

Smoking, hypertension, coronary artery disease, COPD, and chronic renal insufficiency are noted to be risk factors for Leriche syndrome.^2^–^3^ In even rarer occurrences, AOD can present in a person younger than 50 years old. In these younger patients, there is a higher incidence of hypercoagulopathy such as in antiphospholipid syndrome (APS).^2^–^4^

It should be noted that this disease could be confused with acute spinal myelopathy, particularly since patients will often complain of paraplegia and paresthesia of the lower extremities. To further differentiate aortoiliac occlusion from spinal myelopathy, the patient’s history will often reveal a complaint of intolerable pain in the legs, similar to our case patient. Diminished pulses, pallor, and decreased temperature are also more indicative of vascular compromise.^5^

The largest modern case series of Leriche syndrome was performed at Oregon Health and Science University with 29 patients. Most of their patients were male with a median age at diagnosis of 60.7 years, smokers, and diagnosed with hypertension, peripheral artery disease, and coronary artery disease. Five of the patients were predisposed to Leriche syndrome due to their hypercoaguability from APS.^3^–^5^ Seven of the patients had known malignancy. Their findings showed that patients who were over the age of 60 years, smokers, had motor and sensory deficits at the time of presentation, and had acute aortoiliac occlusion at the level of renal arteries were associated with the worst 30-day mortality rate.^3^

Management of aortoiliac occlusion has classically been open repair or endovascular intervention. For patients with critical limb ischemia and risk of future amputation, stents or open repair is acceptable. Thrombolysis and mechanical thrombectomy have been used as an adjunct in angioplasty and stenting. Axillobifemoral bypass also provides excellent short-term patency.^2^

Our patient exhibited the clinical features of pallor, mottled skin, pulselessness, paresthesia, and bilateral paraplegia of the lower extremities. These are unfortunately late manifestations of Leriche syndrome. We suspect his complaint of worsening chronic back pain was actually related to his not-yet-discovered Stanford Type B dissection. His AOD then manifested as new onset bilateral leg pain and paraplegia. His gender, age, smoking history, hypertension and COPD were all noted to be risk factors in developing AOD.^5^–^8^

High mortality rates in AOD are associated with cardiac complications, older patients, and COPD. In this case, the patient had multi-system organ failure along with a chronic Stanford Type B aortic dissection (Image 1). The physical examination demonstrated a complete lack of vascular perfusion in the bilateral lower extremities. Paralysis of the bilateral lower extremities in this case was likely due to hypoperfusion of the spinal arteries. Renal failure and rhabdomyolysis were likely inevitable due to the thrombosis and near-complete occlusion of the infrarenal arteries. The poorly perfused lower extremities eventually led to myonecrosis of the lower extremities, which further contributed to the rhabdomyolysis and renal failure (Image 3). The patient’s use of naproxen and aspirin may have exacerbated his renal failure as well. Unfortunately, this patient succumbed to severe limb ischemia and multi-system organ failure. While this is not the first case of Leriche syndrome causing multi-system organ failure, this case is the first noted presentation of a Stanford Type B thoracic aortic dissection associated with aortoiliac occlusion.

## CONCLUSION

Leriche syndrome is a rare and extremely severe form of peripheral arterial disease whose clinical findings of vascular compromise manifest late in the course of the disease. While AOD remains associated with high morbidity and mortality, immediate recognition and operative management have shown to improve outcomes. The emergency physician should be aware of the demographics and risk factors associated with these patients to ensure prompt treatment.
